# Representations of minimum unit pricing for alcohol in UK newspapers: a case study of a public health policy debate

**DOI:** 10.1093/pubmed/fdu078

**Published:** 2014-10-13

**Authors:** Chris Patterson, Srinivasa Vittal Katikireddi, Karen Wood, Shona Hilton

**Affiliations:** MRC/CSO Social and Public Health Sciences Unit, University of Glasgow, Glasgow, UK

**Keywords:** alcohol, policy, media, content analysis

## Abstract

**Background:**

Mass media influence public acceptability, and hence feasibility, of public health interventions. This study investigates newsprint constructions of the alcohol problem and minimum unit pricing (MUP).

**Methods:**

Quantitative content analysis of 901 articles about MUP published in 10 UK and Scottish newspapers between 2005 and 2012.

**Results:**

MUP was a high-profile issue, particularly in Scottish publications. Reporting increased steadily between 2008 and 2012, matching the growing status of the debate. The alcohol problem was widely acknowledged, often associated with youths, and portrayed as driven by cheap alcohol, supermarkets and drinking culture. Over-consumption was presented as a threat to health and social order. Appraisals of MUP were neutral, with supportiveness increasing slightly over time. Arguments focused on health impacts more frequently than more emotive perspectives or business interests. Health charities and the NHS were cited slightly more frequently than alcohol industry representatives.

**Conclusion:**

Emphases on efficacy, evidence and experts are positive signs for evidence-based policymaking. The high profile of MUP, along with growing support within articles, could reflect growing appetite for action on the alcohol problem. Representations of the problem as structurally driven might engender support for legislative solutions, although cultural explanations remain common.

## Introduction

Mass media influence which issues are presented to the public, and how they are represented.^1^ In a process called framing, media construct problems, causes and solutions by selectively presenting issues, choosing which components to mention or omit.^2–4^ By setting agendas and creating frames, media influence public understandings.^1^ Understanding framing may illuminate how public health policy debates play out in the media.

Alcohol contributes to health risks, social harms and economic burdens.^5^ The United Kingdom's (UK) consumption has outpaced other western European countries, matched by declining health.^6^ In the UK the Scottish, Northern Irish and Welsh administrative branches determine health policy within those regions, while the UK Government controls English health policy. Both the Scottish and UK governments have identified the need to tackle the alcohol problem,^7,8^ and the role of legislation in doing so.

Affordability is known to drive alcohol purchasing, consumption and harm.^9^ Minimum unit pricing (MUP) is an intervention designed to reduce alcohol purchasing and consumption by setting a uniform minimum price below which no unit of alcohol may be sold. Policymakers may be emboldened by the successes of smoke-free legislation, support for which increased following implementation.^10,11^ Econometric analyses^12^ and evaluations of comparable interventions outside the UK^13,14^ suggest that price increases can reduce consumption and harms. UK consumers are consciously price-sensitive,^15^ but some express concerns that MUP would unfairly affect moderate and low-income drinkers.^16^ Conversely, modelling evidence suggests that the intervention would primarily affect harmful, low-income drinkers, with little impact on moderate drinkers.^17^ Australian research identified public scepticism about disrupting alcohol culture with policy.^18^

The Scottish Parliament passed the Alcohol (Minimum Pricing) (Scotland) Bill into legislation in May 2012, but implementation is currently obstructed by legal challenges from the Scotch Whisky Association.^19^ In March 2009, the UK Chief Medical Officer (CMO) recommended a 50p minimum price per unit for England and Wales.^20^ The UK Government announced intent to introduce MUP in 2012,^21^ but confirmed in July 2013 that they had reneged, with some observers suggesting evidence had been ignored due to alcohol industry influence.^22,23^ Evidence suggests that the policy community in post-devolution Scotland is less accommodating to industry lobbying than its UK counterpart, having disrupted relationships between industry representatives and policymakers.^24,25^ This may partially explain the differing fates of MUP in each government, although broader institutional and political factors may play their roles.^26^ Analyses of evidence submitted to the Scottish Government's 2008 consultation into tackling the alcohol problem suggests that industry sources misrepresented evidence to strengthen their case against MUP,^27^ and industry interests highlighted differing objectives for alcohol policy than non-industry actors.^28^

Literature about media coverage of alcohol largely focuses on advertising or entertainment content.^29^ Those focusing on news largely analyse US^30^ or Australian^31^ sources. Nicholls^32^ studied alcohol reporting in UK newspaper and television news, examining articles from two brief time periods, including images and advertisements.

We studied newspaper news coverage of MUP as a case study of how mass media public health policy debates develop. To our knowledge, this study is the first examining representations of a specific alcohol policy debate throughout an extended period, and the first quantitative analysis of UK newsprint coverage of MUP. We offer new insight by comprehensively analysing representations of MUP and alcohol-related issues in UK newspaper news articles. This research was conducted alongside two analyses of qualitative data from a sub-sample of articles. One focused on representations of, and contributions from, key advocates and critics of MUP,^33^ while the other examined representations of the harms posed by alcohol to ‘others’.^34^

## Method

To understand UK national newspaper news coverage of MUP, we employed methods of sampling, data collection and analysis established in prior media content analysis studies.^35–38^ A sample of publications was selected purposively to be diverse in terms of regional perspective and readership profile, and each publication had high circulations (Table 1). Three Scottish national newspapers and seven UK national newspapers were selected, representing three genres: tabloid, middle-market tabloid and serious. This typology has been used in previous UK newspaper content analyses,^38–40^ and ensured the sample largely represented the breadth of UK national newspaper coverage of the issue. Online editions were excluded.

**Table 1:** Summary of publications and articles in sample

*Title*	*Circulation^a^*	*Total articles*		*Front page articles*		*Word count*		
		**n**	*%*	**n**	*%*	*First quartile*	*Median*	*Third quartile*
UK								
Serious								
Guardian & The Observer	2 781 000	42	4.7	0	0	424.0	545.5	715.0
Independent & Independent on Sunday	2 607 000	26	2.9	0	0	593.0	936.0	1176.0
Daily Telegraph & Sunday Telegraph	3 051 000	65	7.2	12	18.5	352.0	504.0	652.0
Middle-market								
Daily Mail & Mail on Sunday	9 521 000	35	3.9	2	5.7	413.0	593.0	763.0
Express & Sunday Express	2 683 000	101	11.2	1	1.0	227.0	347.0	481.0
Tabloid								
Mirror & Sunday Mirror	6 762 000	22	2.4	0	0	152.0	239.0	490.0
The Sun & News of the World	12 400 000^b^	126	14.0	2	1.6	124.0	195.0	377.0
Scotland								
Serious								
The Herald & The Sunday Herald	296 000	206	22.9	16	7.8	313.0	507.0	635.0
Scotsman & Scotland on Sunday	334 000	106	11.8	2	1.9	429.0	528.0	790.5
Tabloid								
Daily Record & Sunday Mail	1 503 000	172	19.1	18	10.5	154.0	243.5	399.0
Total		901	100	53	5.9	240.0	475.6	626.0

^a^Estimated weekly readership from the National Readership Survey, August 2013 (http://www.nrs.co.uk).

^b^Circulation figures for The Sun & The Sun on Sunday; The Sun on Sunday replaced the News of the World in February 2012.

Researchers searched the *Nexis UK* and *Newsbank* databases for articles containing variants of the terms ‘alcohol’ and ‘pricing’ published between 1 January 2005 and 30 June 2012. The period begins before Scottish Health Action on Alcohol Problems' (SHAAP) first endorsement of MUP, and ends following parliamentary passage of the Alcohol (Minimum pricing) (Scotland) Bill. In total, 2076 articles were retrieved, read and filtered. Of these, 1175 were excluded on the basis of meeting one or more criteria: article is from an Irish edition; article is from the TV guide, review, sports, travel, weather or readers' letters section; article duplicates a previously accepted article; and MUP is not the main focus. After filtering, 901 articles remained.

To record article content, researchers developed a coding frame. A basic structure was derived from the literature on alcohol and content analysis. Researchers read 100 randomly selected articles, adding emergent themes as thematic codes. Further batches of 20 articles were read until no new codes emerged. This method allows thematic codes to emerge from data organically without requiring pre-defined conceptual frames. The processes of familiarization with data and identifying a thematic framework from both *a priori* and emergent themes are similar to framework analysis.^41^ However, as the textual data in the articles were coded numerically, the resulting analysis was quantitative.

Codes were grouped into categories in the coding frame. Table 2 lists the categories and codes used. Researchers (C.P., K.W.) recorded manifest content, noting when the article text contained overt statements falling within a thematic code. Manifest content is presented overtly, is quantifiable and facilitates analysis of broad trends in large samples, while latent content requires interpretive reading of underlying meanings, facilitating more nuanced qualitative analysis.^42^

**Table 2:** Reporting on the alcohol problem, affected groups, drivers and arguments

	*Publication region*							*Publication genre*						
	*All articles (*n* = 901)*		*Scotland (*n* = 484)*		*UK (*n* = 417)*		*Regression *P*-value^a^*	*Tabloid (*n* = 254)*		*Middle market (*n* = 136)*		*Serious (*n* = 511)*		*Chi-squared *P*-value^b^*
	n	*%*	n	*%*	n	*%*		n	*%*	n	*%*	**n**	*%*	
**Reporting on the alcohol problem**	674	74.8	339	70.0	335	80.3	<0.001***	173	68.1	109	80.1	392	76.7	0.011*
Mentions an alcohol problem within the UK	564	62.6	282	58.3	282	67.6	<0.001***	148	58.3	85	62.5	331	64.8	0.216
Mentions alcohol as a risk to personal health	365	40.5	175	36.2	190	45.6	0.001**^e^	83	32.7	65	47.8	217	42.5	0.006**
Mentions alcohol as a risk to others, society	335	37.2	169	34.9	166	39.8	0.076	80	31.5	57	41.9	198	38.7	0.069
Mentions alcohol as an economic problem	220	24.4	109	22.5	111	26.6	0.185	64	25.2	35	25.7	121	23.7	0.834
Mentions alcohol as a burden on the NHS	124	13.8	43	8.9	81	19.4	<0.001***	26	10.2	23	16.9	75	14.7	0.125
Mentions alcohol as a burden on the police	53	5.9	24	5.0	29	7.0	0.007**	11	4.3	5	3.7	37	7.2	0.135
**Reporting on groups most affected by the alcohol problem**	221	24.5	99	20.5	112	29.3	0.011*	54	21.3	45	33.1	122	23.9	0.002**
Mentions youths in relation to high-risk drinking	189	21.0	84	17.4	105	25.2	0.010*	45	17.7	38	27.9	106	20.7	0.060
Mentions women in relation to high-risk drinking	77	8.6	33	6.8	44	10.6	0.070	20	7.9	15	11.0	42	8.2	0.525
Mentions men in relation to high-risk drinking	55	6.1	25	5.2	30	7.2	0.062	15	5.9	7	5.1	33	6.5	0.841
**Reporting on the drivers of the alcohol problem**	686	76.1	356	73.6	330	79.1	0.055	183	72.0	111	81.6	392	76.7	0.096
Mentions cheap alcohol or ‘problem drinks’	545	60.5	285	58.9	260	62.4	0.023*	137	53.9	81	59.6	327	64.0	0.027*
Mentions a negative drinking culture	359	39.8	184	38.0	175	42.0	0.789	101	39.8	64	47.1	194	38.0	0.157
Mentions supermarkets	259	28.8	119	24.6	140	33.6	0.001***	63	24.8	36	26.5	160	31.3	0.141
Mentions drinks promotions, happy hours etc.	259	28.8	136	28.1	123	29.5	0.287	64	25.2	39	28.7	156	30.5	0.308
Mentions alcohol advertising or marketing	91	10.1	38	7.9	53	12.7	0.002**	16	6.3	17	12.5	58	11.4	0.055
**Framing arguments for and against MUP**														
MUP is supported by experts/stakeholders	471	52.3	252	52.1	219	52.5	0.069	119	46.9	63	46.3	289	56.6	0.013**
MUP would be effective	413	45.8	227	46.9	186	44.6	0.339	117	46.1	47	34.6	249	48.7	0.013**
MUP is not supported by experts/stakeholders	367	40.7	217	44.8	150	36.0	0.741	72	28.3	45	33.1	250	48.9	0.001***
MUP would be ineffective	349	38.7	182	37.6	167	40.1	0.298	81	31.9	59	43.4	209	40.9	0.026*
MUP will punish responsible drinkers/the poor	288	32.0	128	26.5	160	38.4	<0.001	70	27.6	51	37.5	167	32.7	0.116
There is evidence to support MUP	257	28.5	135	27.9	122	29.3	0.012*	49	19.3	34	25.0	174	34.1	<0.001***
MUP is likely to face legal challenges	252	28.0	156	32.2	96	23.0	0.089	53	20.9	31	22.8	168	32.9	0.001**
MUP is good for public health and/or society	242	26.9	119	24.6	123	29.5	0.027*	56	22.0	40	29.4	146	28.6	0.122
MUP would be bad for business	194	21.5	136	28.1	58	13.9	<0.001	41	16.1	22	16.2	131	25.6	0.003**
There is no evidence to support MUP	174	19.3	99	20.5	75	18.0	0.331	43	16.9	28	20.6	103	20.2	0.522
MUP will increase retailers' revenues	162	18.0	103	21.3	59	14.2	0.014*	48	18.9	18	13.2	96	18.8	0.294
MUP has public support	24	2.7	15	3.1	9	2.2	0.532	8	3.1	2	1.5	14	2.7	0.610
MUP does not have public support	17	1.9	4	0.8	13	3.1	0.065	3	1.2	6	4.4	8	1.6	0.059

^a^Linear regression of the relationship between publication region and mentioning a given theme, controlling for genre.

^b^The Chi-squared test of whether proportions differed between genres.

*P < 0.05.

**P < 0.01.

***P < 0.001.

The only code requiring latent coding was supportiveness of MUP, for which we developed a five-point scale comprising: supportive of MUP; mostly supportive of MUP; neutral/no stance taken on MUP; mostly against MUP; and against MUP. Rather than gauging the journalist's position, supportiveness reflects the frequency of arguments favouring and opposing MUP within each article, presented as either editorial or external perspectives. Articles exclusively containing either supportive or oppositional arguments were coded as ‘supportive’ or ‘against’, respectively. Articles predominantly, but not exclusively, containing positive arguments were coded as ‘mostly supportive’, while articles with the inverse distribution of arguments were coded as ‘mostly against’. Articles containing no arguments, or equal proportions of supportive and unsupportive arguments, were coded as ‘neutral/no stance taken’. Using this measure of supportiveness, even ‘news’ articles comprising relatively factual, non-opinionated reporting could be coded as supportive or unsupportive of MUP. Supportiveness was double-coded on a randomly selected 10% of articles. A linearly weighted kappa test of inter-rater agreement returned a coefficient of 0.87, which can be interpreted as ‘almost perfect’ agreement.^43^

Data were analysed using Stata v10.^44^ Chi-squared tests were used to test how genre and format related to thematic codes. One-sample *t*-tests were used to test how each publication's mean support differed from both the overall sample mean and a neutral level of support. Linear regressions were used to investigate relationships between thematic codes and publication region, and relationships between characteristics of articles and their support for MUP. Where appropriate, regressions were adjusted by word count to account for the proportion of each article focusing on relevant content; longer articles are more likely to include content falling under our thematic categories due to their length, but a short article focused wholly on one aspect of the issue is no less important. Similarly, we adjusted tests of between-publication differences by genre to minimize its potential confounding effect.

## Results

### Overview of articles

Sample publications published 901 articles about MUP between 1 January 2005 and 30 June 2012. Fifty-two (6%) were on front pages, representing a large proportion of coverage; by comparison, 4.7% of articles in a study of reporting on H1N1 influenza were on front pages.^45^ Table 1 details the number of articles, front page articles and the distribution of word counts by publication.

More than half of articles were published in the three Scottish publications (484, 53.7%). Per publication, Scottish newspapers reported on MUP much more than UK newspapers. Most articles were in serious genre publications (511, 56.7%), and most were news format (679, 75.4%).

### Trends in reporting over time

**Fig. 1:**
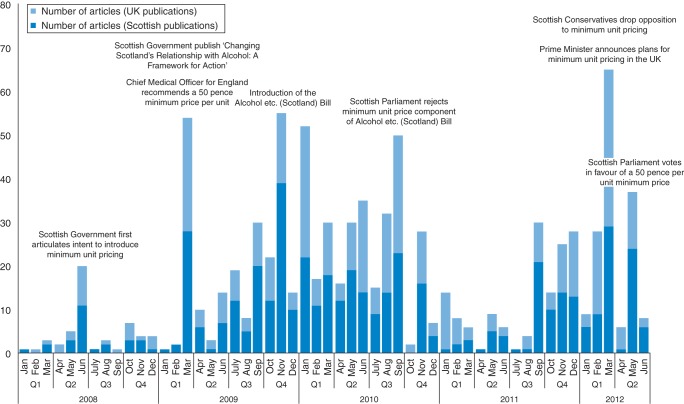
Frequency of articles reporting on MUP by month.

Four articles related to pricing control interventions, but not MUP, were published between 2005 and 2007. Reporting about MUP began in 2008. Frequency of reporting increased month-to-month between January 2008 and June 2012, and varied with news events (Fig. 1 ).

The Scottish Government's policy dominated reporting prior to December 2011. Reporting on the UK Government's policy peaked at 36 articles in March 2012 when the UK Government's alcohol strategy was published,^46^ and fell to six by June 2012.

### Reporting on the alcohol problem

Three-quarters of articles (*n* = 674) overtly mentioned the alcohol problem (see Table 2 for problem definitions and frequencies). When adjusted for word count, middle-market publications were significantly more likely to mention any problem definition and alcohol as a health risk. News format articles were significantly less likely to mention: any alcohol problem; a problem within the UK; a health risk; or a risk to society.

### Reporting on groups affected by the alcohol problem

Youths (‘children’, ‘adolescents’ etc.) were mentioned more than women and men (Table 2). Articles in Scottish publications were significantly less likely to mention specific groups in general, and youths in particular. Similar relationships existed when adjusting for genre. Middle-market publications mentioned youths significantly more frequently. Serious publications were significantly less likely to mention youths when adjusting for word count, and significantly less likely to mention women. Format was significantly related to mentioning youths, women and men; each was mentioned in commentary or feature articles more than news articles.

### Reporting on drivers of the alcohol problem

The most frequently mentioned drivers were cheap alcohol (545, 60.5%), drinking culture (359, 39.8%) and supermarkets (259, 28.8%) (Table 2). Format had significant, positive relationships with mentioning drinking culture and advertising. Culture was mentioned in commentary and feature articles more commonly than news articles. Advertising was mentioned more commonly in commentary articles.

### Article support for MUP

Articles were approximately neutral towards MUP (mean supportiveness 51.4%), with little difference between regions. Middle-market publications were significantly less supportive than other genres, and commentary articles significantly less supportive than other formats (Table 3).

**Table 3:** Article support for MUP by publication region, genre and format

	**n**	*Mean support for MUP*	**T*-test (difference from sample mean)*	**T*-test (difference from 50%)*
Publication region				
Scotland	484	51.9%	*P* = 0.774	*P* = 0.228
UK	417	50.9%	*P* = 0.760	*P* = 0.591
Publication genre				
Tabloid	254	53.0%	*P* = 0.498	*P* = 0.193
Middle-market	136	42.3%	*P* = 0.003**	*P* = 0.012*
Serious	511	53.1%	*P* = 0.244	*P* = 0.032*
Publication format				
Commentary	152	43.9%	*P* = 0.028*	*P* = 0.074
News	679	52.3%	*P* = 0.473	*P* = 0.059
Feature	70	59.3%	*P* = 0.083	*P* = 0.041*

*P < 0.05.

**P < 0.01.

***P < 0.001.

A linear regression indicated that supportiveness increased significantly by an average of 0.2% per month across the sample period (*P* = 0.017). In Scottish publications, the increase was 0.4% per month (*P* = 0.001). UK publications exhibited no significant increase. The greatest change within a publication was in the *Scotsman*, with a significant monthly increase of 0.5% (*P* = 0.005). Supportiveness was positively and significantly related to mentioning: any description of the alcohol problem; alcohol as a health risk; alcohol as a risk to society; any driver of the alcohol problem; cheap alcohol; supermarkets; women; and men.

### Framing arguments for and against MUP

Table 2 lists arguments for and against MUP. Arguments involving efficacy, expert support and evidence were most frequent. Few articles mentioned public support (24, 2.7%), or lack of support (17, 1.9%). Controlling for genre, Scottish publications referred to MUP harming business and increasing retailer's revenue significantly more than UK publications, and referred to MUP punishing responsible drinkers or the poor, being supported by evidence or being good for public health significantly less.

Commentary articles were significantly less likely to characterize MUP as: supported by experts or stakeholders; effective; lacking support from experts or stakeholders; or good for public health. News format articles were significantly more likely to characterize MUP as: likely to face legal challenges; good for public health; and bad for business, and significantly less likely to report that MUP would be ineffective. Feature articles were most likely to mention that MUP would be effective and is supported by evidence.

### Stakeholder opinions

Quotations and other references to stakeholders, along with their reported stance towards MUP, are reported in Supplementary Table S1. Politicians were cited most frequently (735, 81.6%), particularly SNP (633, 70.6%), followed by health charities and the NHS (334, 37.1%), and alcohol producers (306, 34.0%). The most frequently referenced supermarket spokesperson was cited 27 (3%) times. Health charities and the NHS were overwhelmingly presented as supportive of MUP, while drinks industry representatives were predominantly opposed.

## Discussion

### Main findings of this study

This study describes UK and Scottish newsprint representations of the MUP policy debate, which had a high profile in both, particularly Scottish. Coverage increased over time, mirroring the progress of the wider debate. Most articles discussed the alcohol problem, predominantly characterizing it in terms of health and social order, often associated with children or youths, and driven by cheap alcohol and drinking culture. Articles were, on aggregate, neutral towards MUP. Support increased over time, mirroring a policy landscape wherein the Scottish Conservative Party and Scottish Liberal Democrats reversed their opposition and the UK Government resolved to introduce MUP. Frequently cited stakeholders included politicians, health charities and industry representatives. Health charities and the NHS were presented as overwhelmingly supportive, and drinks industry stakeholders as almost as uniformly opposed, highlighting division between, and consistency within, these groups. The most frequent arguments concerned efficacy and the support of experts and evidence, as well as perceived injustice towards poor and responsible consumers. Public support and effects on businesses were discussed relatively infrequently.

### What is already known on this topic

Media representations of tobacco policy debates have been studied extensively,^35,47,48^ but little research explores representations of alcohol policies. Some research examines relationships between media and alcohol problems,^30,31,49,50^ but not specific policies. Audience reception research suggested that news consumers may be sceptical about the ability of policy to influence culture, and that they may not readily perceive interventions such as MUP as part of a broad package of policies.^18^

Our findings support those of Nicholls,^32^ who identified politicians, health charities and the alcohol industry as the most cited stakeholders in the MUP debate, and found that articles associated cheap alcohol and supermarkets with excessive consumption. Our findings are complemented by our qualitative analyses of newspapers representations of: the key claim-makers in the debate^33^; and the harms caused to ‘others’ by alcohol.^34^ The former examines differences and similarities between opponents and supporters of MUP within the media debate, offering suggestions of how evidence-based public health policy might be better advocated in the media,^33^ while the latter examines representations of the social harms that alcohol may cause, drawing conclusions about how those representations might influence public acceptance of population-based solutions.^34^

### What this study adds

Advocates will welcome MUP's high profile and some characteristics of the coverage. Articles problematize alcohol primarily in terms of health and social order, characterizations that have been prioritized by the Scottish and UK governments.^7,8^ Associations between the different national debates and different characterizations were not evident, but articles mentioning health risks tended to be more supportive than those mentioning social disorder.

The association of children and youths with alcohol problems could have implications for the framing of solutions, as constructions of affected societal groups can influence appraisals of solutions.^28,51,52^ Associating children, a powerless but positively constructed social group, with the alcohol problem could stimulate support for legislative solutions. Conversely, some categories of ‘young people’ may be viewed as ‘deviants’^51^ engaged in individual-level misbehaviour to which top-down solutions might seem ill-suited. Audience reception research might investigate how associations of alcohol problems with children influence perceptions of solutions.

Presentations of problem drivers can influence appraisals of solutions,^28,52^ so it is appropriate to consider the potential implications of how drivers of the alcohol problem were depicted. Frequent reporting of cheap drinks, supermarkets and promotions may contribute to a structural causal frame suited to structural solutions. Cultural drivers are more complex; while readers may believe legislative change can mediate culture, culture is often perceived as slow-changing and resistant to discrete legislative solutions. Australian evidence suggests news audiences view ‘drinking culture’ as a more powerful driver than price, and doubted legislation's ability to influence culture.^18^ We found no relationship between mentioning drinking culture and support for MUP. Audience reception research could improve understandings of associations between perceptions of drivers and attitudes towards solutions.

We found that articles were neutral towards MUP overall, and supportiveness increased over time. Increased media support may be mirrored by increased public support through gradually increasing familiarity with MUP, as was the case with smoke-free legislation.^11^ The predominance of arguments related to efficacy, evidence and expert support was consistent with the evidence-based policy, suggesting the media debate largely focused on health impacts instead of emotive perspectives or business interesting, and that industry interests did not take precedence over health charities. A debate focused on efficacy, evidence and experts echoes calls for evidence-based policymaking, but is not necessarily evidence of a substantive shift in favour of evidence-based policy.

News format articles were more supportive than commentary, feature or editorial articles. This difference may hold lessons for advocates; public health advocates might benefit from better representation in non-news formats, perhaps by engaging a broader range of journalists beyond health writers, or seeking more opportunities to write as guest contributors.

In addition to our concurrent qualitative analyses,^33,34^ our research could benefit from further investigation. Further research could focus on societal groups associated with the alcohol problem, determining how different sub-groups of ‘young people’ are constructed, comparing constructions of men and women or analysing constructions of different categories of ‘problem’ drinkers. Further content analyses might also examine media beyond newsprint.

### Limitations of this study

Quantitative content analysis allows overviews of manifest content of large samples, but is not suited to investigating specific elements of frames in depth or analysing context in detail, and cannot determine authors' intentions or audiences' interpretations.^53^ In this research, scope for comparative analysis of representations of the UK and Scottish debates was limited as few articles discussed the UK Government's proposed policy. Additionally, it should be noted that comparisons of UK and Scottish newspapers are not straightforward comparisons between two discrete regions' exclusive publications, rather UK publications are written partly for Scottish readers, and also publish Scottish editions containing articles tailored for that audience. More generally, the focus on newspapers precludes investigation of representations within other media, which are increasingly relevant as newspaper circulation declines.^54^ Methodologically, this research would be more robust if every article were double-coded; double-coding the latent content of a random 10% sub-sample indicated high agreement, but comprehensive double-coding would have been optimal.

**Key points**

- MUP has been a high-profile issue in UK and Scottish newspapers
- Arguments about MUP policy tended to focus on what works to improve health outcomes, rather than focusing on emotive perspectives or the interests of business
- The alcohol problem was presented as driven by cheap alcohol and a negative drinking culture
- Appraisals of the intervention were neutral overall, but supportiveness increased over time
- Presentations of the problem and its drivers may contribute to a structural causal frame, depicting the problem as one suited to structural, legislative solutions

## Supplementary data

Supplementary data are available at the *Journal of Public Health* online.

## Conflict of interest

S.H. and S.V.K. are investigators planning an evaluation of MUP. Several years ago, S.V.K. received payment for writing opinion articles for the Scotsman newspaper, but has never written about the topic of alcohol in the mass media. The authors declare they have no other conflict of interest.

## Supplementary Material

Supplementary Data
