# Supplementary material for: Representations of minimum unit pricing for alcohol in UK newspapers: a case study of a public health policy debate
Source: J Public Health (Oxf). 2014 Oct 13;37(1):40–9. doi: 10.1093/pubmed/fdu078 (PMC4340327; doi:10.1093/pubmed/fdu078)
Supplement: Supplementary Data [file supp_37_1_40__index.html]

Representations of minimum unit pricing for alcohol in UK newspapers: a case study of a public health policy debate — Representations of minimum unit pricing for alcohol in UK newspapers: a case study of a public health policy debate — Supplementary Data 

# Representations of minimum unit pricing for alcohol in UK newspapers: a case study of a public health policy debate

## Supplementary Data

Supplementary Data

**Files in this Data Supplement:**

- Supplementary Table 1 - doc file
