# Supplementary material for: Representations of minimum unit pricing for alcohol in UK newspapers: a case study of a public health policy debate
Source: J Public Health (Oxf). 2014 Oct 13;37(1):40–9. doi: 10.1093/pubmed/fdu078 (PMC4340327; doi:10.1093/pubmed/fdu078)
Supplement: Supplementary Data [file supp_fdu078_fdu078supp_table1.doc]

| Stakeholder type | Any mention | | Mentioned as supportive of MUP | | Mentioned as unsupportive of MUP | | News articles | | Commentary and editorial articles | | Feature articles | |
| --- | --- | --- | --- | --- | --- | --- | --- | --- | --- | --- | --- | --- |
|  | n | % | n | % | n | % | n | % | n | % | n | % |
| Politicians (any) | 735 | 81.6 | 694 | 77.0 | 450 | 49.9 | 573 | 63.6 | 108 | 12.0 | 54 | 6.0 |
| Politicians (Scottish National Party) | 633 | 70.3 | 633 | 70.3 | 0 | 0.0 | 501 | 55.6 | 86 | 9.5 | 46 | 5.1 |
| Health charities/NHS | 334 | 37.1 | 333 | 37.0 | 1 | 0.1 | 258 | 28.6 | 51 | 5.7 | 25 | 2.8 |
| Politicians (Conservative) | 312 | 34.6 | 87 | 9.7 | 259 | 28.7 | 253 | 28.1 | 44 | 4.9 | 15 | 1.7 |
| Politicians (Labour) | 311 | 34.5 | 35 | 3.9 | 304 | 33.7 | 252 | 28.0 | 36 | 4.0 | 23 | 2.6 |
| Drinks industry | 306 | 34.0 | 45 | 5.0 | 276 | 30.6 | 254 | 28.2 | 37 | 4.1 | 16 | 1.8 |
| Politicians (Liberal Democrat) | 158 | 17.5 | 49 | 5.4 | 121 | 13.4 | 127 | 14.1 | 22 | 2.4 | 9 | 1.0 |
| Off-licence retailers and representatives | 106 | 11.8 | 28 | 3.1 | 90 | 10.0 | 86 | 9.5 | 12 | 1.3 | 8 | 0.9 |
| Police | 100 | 11.1 | 100 | 11.1 | 0 | 0.0 | 71 | 7.9 | 15 | 1.7 | 14 | 1.6 |
| Alcohol charities | 95 | 10.5 | 92 | 10.2 | 4 | 0.4 | 78 | 8.7 | 8 | 0.9 | 8 | 0.9 |
| Bar and club owners and representatives | 51 | 5.7 | 44 | 4.9 | 10 | 1.1 | 37 | 4.1 | 5 | 0.6 | 9 | 1.0 |
| Economists | 50 | 5.5 | 9 | 1.0 | 41 | 4.6 | 42 | 4.7 | 5 | 0.6 | 3 | 0.3 |
| Supermarket: Tesco | 27 | 3.0 | 24 | 2.7 | 5 | 0.6 | 17 | 1.9 | 6 | 0.7 | 4 | 0.4 |
| Supermarket: ASDA | 17 | 1.9 | 0 | 0.0 | 17 | 1.9 | 16 | 1.8 | 1 | 0.1 | 0 | 0.0 |
| Supermarket: Sainsbury's | 10 | 1.1 | 0 | 0.0 | 10 | 1.1 | 2 | 0.2 | 0 | 0.0 | 2 | 0.2 |
| Supermarket: other | 6 | 0.7 | 1 | 0.1 | 5 | 0.6 | 6 | 0.7 | 0 | 0.0 | 0 | 0.0 |
| Supermarket: Morrison's | 4 | 0.4 | 0 | 0.0 | 4 | 0.4 | 3 | 0.3 | 0 | 0.0 | 1 | 0.1 |
